# Buccolingual Inclination of Canine and First and Second Molar Teeth and the Curve of Wilson in Different Sagittal Skeletal Patterns of Adults Using Cone-Beam Computed Tomography

**DOI:** 10.1155/2020/8893778

**Published:** 2020-10-31

**Authors:** Amin Golshah, Navid Rezaei, Sara Heshmati

**Affiliations:** ^1^Department of Orthodontics, Faculty of Dentistry, Kermanshah University of Medical Sciences, Kermanshah 6715847141, Iran; ^2^Students Research Committee, Faculty of Dentistry, Kermanshah University of Medical Sciences, Kermanshah 6715847141, Iran

## Abstract

**Objectives:**

This study aimed to assess the buccolingual inclination of canine and first and second molar teeth and the curve of Wilson in different sagittal skeletal patterns in untreated adults using cone-beam computed tomography (CBCT).

**Materials and Methods:**

Sixty-six CBCT scans of adults (mean age: 28.74 ± 5.25 years) were evaluated in this cross-sectional study. The images were standardized using the Frankfurt horizontal plane and the interorbital line. The sagittal skeletal pattern was determined using the ANB angle and Wits appraisal. Inclination angles were measured by NNT Viewer and Mimics software. The curve of Wilson was measured by connecting the tips of mesiobuccal and mesiolingual cusps of maxillary first and second molars along the buccal groove and measuring the formed angle. Data were analyzed using ANOVA.

**Results:**

The intraobserver agreement was 0.969. The mean inclination of maxillary first and second molars in class I and III patients was significantly higher than that in class II patients (*P* < 0.05). The mean inclination of mandibular first and second molars in class II patients was significantly higher than that in class I and III patients (*P* < 0.05). The difference in inclination of maxillary and mandibular canine teeth was not significant (*P* > 0.05). The mean curve of Wilson in second molars of class II patients was significantly higher than that in class I patients (*P* < 0.05).

**Conclusion:**

In different sagittal skeletal patterns, a compensatory relationship exists between the opposing teeth, which, along with the standards of crowns, can be used to determine the appropriate position of teeth in dental arch.

## 1. Introduction

Orthodontics is the art and science of leveling and alignment of the teeth in dental arch, which is associated with bone remodeling and converts a malocclusion into a stable occlusion with maximum achievable order and alignment of teeth and optimal function and esthetics. Orthodontic appliances are used for this purpose [1]. Occlusion is a fundamental part of orthodontic treatment [2–6]. Different theories have proposed different definitions for a normal occlusion [7, 8]. However, the clinical application of concepts of occlusion has not been well studied. For instance, the role of buccolingual position of the posterior cusps of molar teeth in the frontal view in occlusion has not been clearly understood considering the fact that their occlusal surfaces do not follow the same pattern. In 1911, George Wilson explained this phenomenon by a compensatory curve to prevent the possible balancing interferences. This curve should be concave in the mandibular arch and concave or convex in the maxillary arch. Thus, the buccal and palatal cusps of the posterior teeth are in functional contact with each other [9].

From the frontal view, the occlusal plane is in the form of an arch. The occlusal surfaces of maxillary posterior teeth comprise the convex part of the arch while the occlusal surfaces of the mandibular posterior teeth comprise the concave part of the arch [10]. Recently, the occlusal inclination was defined as a progressive increase in axial inclination of molars from the first molar to the third molar, which is a developmental feature known as the helicoid curve [11, 12].

From the frontal view, this curve includes the buccal inclination of maxillary molars and the lingual inclination of the mandibular molars. However, it should be noted that the exact amount of this inclination has not yet been quantified [13].

The buccolingual inclination of teeth has long been an interesting topic for orthodontists. Andrews described the six keys to a normal occlusion [14], and according to him, the buccolingual inclination of teeth is one of the six keys to a normal occlusion and is part of the third phase of clinical examinations according to the American Board of Orthodontists (ABO) [14, 15]. The third key of Andrews is related to coronal inclination, which is measured at the buccal surface of tooth crown. The findings of Andrews revealed the lingual inclination of the crown of maxillary and mandibular molars. A wide range of values have been reported. Andrews reported a 27° range for the maxillary first molar and 46° range for the mandibular first molar [16]. ABO stated that in order to obtain a suitable occlusion, maximum intercuspation and no balancing interferences, there should be no significant difference between the height of buccal and lingual cusps of maxillary and mandibular molars and premolars. Thus, they tried to find a clinically acceptable level for buccolingual inclination of posterior teeth by comparing the difference in height of buccal and lingual cusps [15]. The fourth key is related to the curve of Wilson, that describes the inclination of maxillary posterior teeth as a concave curve that adjusts the lingual torque of molar teeth [9]. The curve of Wilson is a hypothetical curve that connects the buccal and lingual cusp tips of the right and left molar and premolar teeth [17]. According to the classification system introduced by the ABO, maximum intercuspation without balancing interferences was characterized by a curve between the maxillary molar cusps and the mandibular arch, which is slightly concave. They confirmed that the lingual cusps were 1-2 mm lower than the palatal cusps [9]. Studies on the curve of Wilson are scarce, and the available ones have evaluated the changes in the curve of Wilson during growth and development [18] or palatal expansion [19] or its role as an etiologic factor in development of temporomandibular disorders [20].

Evidence shows that computed tomography (CT) is beneficial for measurement of transverse dimensions [21, 22]. Several studies have used dental casts [23, 24], CT [25, 26], and cone-beam computed tomography (CBCT) [9, 27, 28] for assessment of inclination of teeth and the mechanics of treatment. For instance, Tsunori et al. [25] showed that facial type (which is correlated with the masticatory function) had a correlation with buccolingual inclination of first and second molars. CBCT now enables more accurate visualization of anatomical structures and easier detection of pathologies.

CBCT is commonly used in dentistry due to low exposure dose (compared with CT) and high resolution, and is frequently requested for implant and orthodontic treatment planning. CBCT has a slice-by-slice mode that enables the visualization of each tooth in any desired plane [29, 30]. Moreover, CBCT enables the evaluation of the entire tooth structure. Thus, uncertainties in the longitudinal axis (inclination) of the teeth due to the use of casts with asymmetrical wear of cusps or tooth morphology are eliminated [1, 9, 26, 27]. Several techniques have been used to determine the longitudinal axis and measure the inclination of teeth such as CT [26] and CBCT [9, 27, 30]. CBCT can be used to assess the position of teeth in the sagittal, axial, and coronal planes.

There is a gap of information regarding the buccolingual inclination of second molars in untreated adults [13, 27]. Change in buccolingual inclination of teeth is an important factor affecting the stability of dentition [27]. Considering the significance of second molars in orthodontic treatment planning and orthosurgery, and the gap of information about the inclination of molar and canine teeth and the curve of Wilson in class I, class II, and class III patients, this study aimed to assess the buccolingual inclination of first and second molars and canine teeth and the curve of Wilson in different sagittal skeletal patterns of untreated adults using CBCT.

## 2. Materials and Methods

This cross-sectional study evaluated the CBCT scans of the maxilla and mandible of adults between 18 and 35 years (both males and females) retrieved from the archives of a radiology clinic. The study was approved in the ethics committee of Kermanshah University of Medical Sciences (IR.KUMS.REC.1397.525). A written informed consent was obtained from all patients.

Sample size was calculated to be 66 records (*n* = 22 in each group) according to a study by Shewinvanakitkul et al. [27] assuming the standard deviation of canine inclination in class I and class II patients to be 3.6 and 4.5, respectively, accuracy (*d*) of 0.5, alpha = 0.05, and power of 90%.

All CBCT scans had been obtained with NewTom VGi CBCT system (Verona, Italy) for orthodontic or surgical treatment planning. The inclusion criteria were CBCT scans taken with 15 × 15 cm field of view in natural head position and maximum intercuspation. The CBCT scans were selected using convenience sampling.

CBCT scans of patients with a history of orthodontic treatment, orthognathic surgery, craniofacial syndromes such as the cleft lip or palate, facial asymmetry, hemihypertrophy of the mandible, pathologies involving the upper airways, upper airway infection, chronic mouth breathing, permanent snoring, history of trauma, missing of more than 4 teeth in each jaw, tonsillar hypertrophy, adenoids, history of tonsillectomy, and respiratory problems were excluded. CBCT scans on which the critical cephalometric landmarks could not be identified were also excluded.

The CBCT images were evaluated in axial, sagittal, and frontal views. The axial view was used to assess the cross section of teeth. The frontal view was used to assess the transverse pattern of the jaws, inclination of teeth and the curve of Wilson, and the sagittal view was used to assess the anteroposterior relationship and the vertical relationship of the jaws.

**Figure 1:**
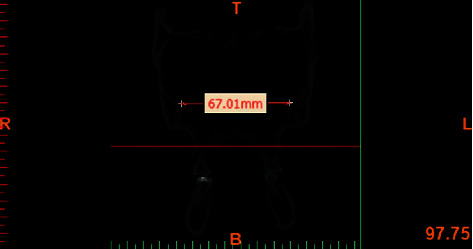
Reorientation of records using the Frankfurt horizontal line and the interorbital line.

All CBCT images were obtained with 300 *μ*m spatial resolution, 110 kV and 78.59 mAs. The CBCT data were exported in DICOM format using NNT Viewer software. The Mimics Medical Software (version 19, Materialise, Leuven, Belgium) was used to reconstruct lateral and posteroanterior cephalograms. In order to standardize the images and minimize errors in measurements, all images were reoriented using NNT Viewer Reorientation software (version 19, Materialise, Leuven, Belgium) such that the Frankfurt horizontal plane and the interorbital line (a line connecting the inferior points of the orbital rims) were parallelized to the horizontal line. By doing so, the head position was standardized in all records and all angles were measured based on this line (Figure 1). For cephalometric analysis, the following hard tissue reference points were identified:

  Orbitale (Or): the most inferior point of the orbital rim.
  CI: the incisal edge of canine.
  CA: the apex of canine.
  MO: the central point of the buccolingual width of the occlusal surface of molar tooth.
  MC: the central point of the buccolingual width of the cervical part of the anatomical crown.
  MBM1/MBM2: the mesiobuccal cusp tip of the maxillary first and second molars.
  MLM1/MLM2: the mesiolingual cusp tip of the maxillary first and second molars.

The cephalometric indices used for assessment of the sagittal pattern included the ANB angle and the Wits appraisal; according to which, the samples were divided into class I (ANB: 0–4°; Wits 0 to −1), class II (ANB > 4°; Wits > 0), and class III (ANB < 0°; Wits < −1) groups.

**Figure 2:**
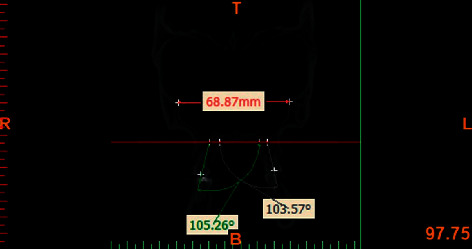
Measuring the inclination of maxillary molars.

**Figure 3:**
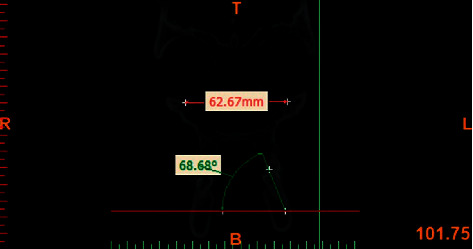
Measuring the inclination of mandibular molars.

**Figure 4:**
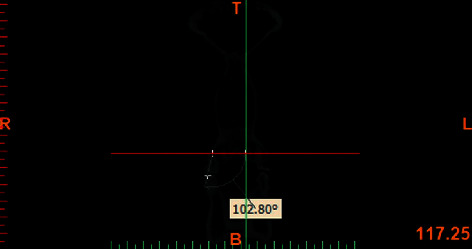
Measuring the inclination of maxillary canines.

**Figure 5:**
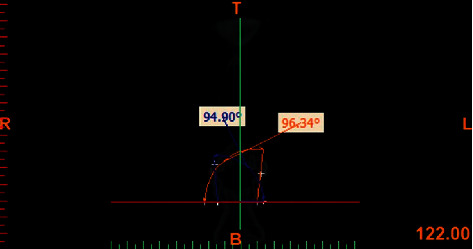
Measuring the inclination of mandibular canines.

The measurements of inclination angles and the curve of Wilson were made using NNT Viewer and Mimics software. In assessment of the buccolingual inclination of molars, the MO point was used as the reference point in order to eliminate the effect of morphology of the cusp of molars. The MC point was used as the reference point to eliminate the effect of root morphology. On the frontal view, the MO-MC line was drawn to determine the buccolingual inclination of posterior teeth (Figures 2 and 3). The CI-CA line was drawn to determine the inclination of canine teeth (Figures 4 and 5). Its angle in the maxilla and mandible was determined by drawing a line parallel to a line connecting the two orbitale points.

**Figure 6:**
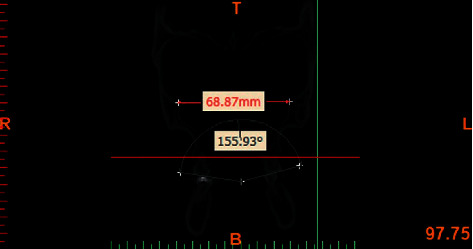
Measuring the curve of Wilson.

In order to measure the curve of Wilson, the mesiobuccal and mesiolingual cusp tips of the maxillary first and second molars were connected along the buccal groove and the formed angle was measured (Figure 6).

Measurements made by an examiner and an experienced radiologist on 20 CBCT scans were repeated again after 2 weeks to assess the intraexaminer reliability. The lowest intraclass correlation coefficient was 0.969, which was considered excellent according to Cicchetti's classification [31]. The Dahlberg's formula was used to assess the method error. The maximum value was found to be 1.01.

Normal distribution of data was evaluated using the Kolmogorov–Smirnov test. The chi-square test was used to compare the study groups in terms of gender. Since data were normally distributed, ANOVA was applied for statistical analysis. For variables with nonhomogeneity of variances, the Welch ANOVA was used. Tukey's post hoc test was applied for pairwise comparisons. All statistical analyses were carried out using SPSS version 18 (SPSS Inc., IL, USA) at 0.05 level of significance.

## 3. Results

A total of 66 records were evaluated; out of which, 34 (51.5%) belonged to females and 32 (48.5%) belonged to males. The mean age of patients was 28.74 ± 5.25 years.

No significant correlation was noted between the sagittal skeletal pattern and gender (Table 1, chi-square test, *P* =0.991). The Kolmogorov–Smirnov test showed that all quantitative variables had normal distribution (*P*  > 0.05).

Table 2 compares the inclination of teeth among the three sagittal skeletal patterns. A significant difference was noted in the inclination of maxillary right second molar among the three sagittal skeletal patterns (*P*=0.002) such that the mean of this variable in class I and class III patients was significantly higher than that in class II patients. Also, a significant difference was noted in the inclination of maxillary right first molar among different sagittal skeletal patterns (*P* < 0.001) such that the mean of this variable in class I and III was significantly higher than that in class II patients. No significant difference was noted in the inclination of maxillary right canine among different sagittal skeletal patterns (*P*=0.053). No significant difference was noted in inclination of maxillary left canine between different sagittal patterns (*P*=0.149). A significant difference was noted in inclination of maxillary left first molar (*P* < 0.001) such that the mean of this variable in class I and III was significantly higher than that in class II patients. A significant difference was noted in inclination of maxillary left second molar (*P*=0.008) such that the mean of this variable in class I was significantly higher than that in class II patients. The difference in inclination of mandibular second molar was also significant (*P* < 0.001), and this variable in class II patients was significantly higher than that in class I and III patients. The difference in inclination of mandibular left first molar was significant as well (*P*=0.002), and this variable in class II patients was significantly higher than that in class I and III patients. No significant difference was noted in inclination of mandibular left canine tooth among different sagittal patterns (*P*=0.858). The difference in inclination of mandibular right canine was not significant either (*P*=0.658). The difference in inclination of mandibular right first molar was significant (*P* < 0.001) such that the mean of this variable in class II was significantly higher than that in class I and III patients. A significant difference was also noted in inclination of mandibular right second molar (*P* < 0.001) such that class II patients had the highest and class I patients had the lowest mean of this variable with significant differences between all three classes.

Table 3 compares the curve of Wilson among the three sagittal skeletal patterns. A significant difference was noted in the curve of Wilson of mandibular second molars between different sagittal patterns (*P*=0.038) such that the mean of this variable in class II patients was significantly higher than that in class I patients. No significant difference was noted in the curve of Wilson of mandibular first molars between different sagittal patterns (*P*=0.253).

Table 4 shows the mean inclination of teeth in the three sagittal skeletal patterns.  Table 5 presents the mean inclination of teeth based on demographic variables of patients.

## 4. Discussion

This study assessed the buccolingual inclination of canine and first and second molar teeth and the curve of Wilson in untreated adults with different sagittal skeletal patterns using CBCT. Of patients, 51.5% were females and 48.5% were males. The results showed no significant association between sagittal skeletal pattern and gender. Patients between 18 and 35 years were included in this study because the inclination of teeth can change during the period of growth and development. Thus, we evaluated patients with completed growth. Marshall et al. [18] reported that maxillary and mandibular molars have lower inclination in adults compared with children. Yang and Chung [32] evaluated 138 patients in three age groups of 6–9, 10–19, and 25–35 years regarding the buccolingual inclination of mandibular and maxillary first molars and concluded that adults have lower buccal and lingual inclination than children. Sayania et al. [33] reported that aging decreases the buccolingual inclination of maxillary and mandibular first molars.

Introduction of CBCT enabled the assessment of inclination of teeth by using the long axis of the teeth instead of their labial surface on the casts, which would increase the accuracy of measurements [9, 13, 27]. Thus, in the present study, to measure the buccolingual inclination of molar teeth, the MO point was used to eliminate the effect of cusp morphology and the MC point was used to eliminate the effect of root morphology. The longitudinal axis to assess the buccolingual inclination of molars was considered as the line that connected the MO to MC. In canines, the line connecting the CI to CA was used for this purpose. Alkhatib and Chung [13] used the entire anatomical crown to determine the longitudinal axis of the teeth. This was done to eliminate any uncertainty due to root deviation or other malformations. The same approach was adopted in the present study. Mitra and Ravi [26] used CT scan to assess the inclination of maxillary molars. They only used the buccal roots for measurement of inclination. Barrera et al. [9] used a line along the central canal to determine the anatomical axis of molar teeth. Shewinvanakitkul et al. [27] drew a hypothetical line along the central canal to the midapex to assess the longitudinal axis of mandibular first molars. In general, it seems that using the entire tooth crown by the help of 3D images yields a more accurate longitudinal axis for assessment of inclination of molar teeth of both jaws [13, 27].

In the present study, for the purpose of standardization, the images were reoriented by the use of NNT Viewer software such that the Frankfurt horizontal line and the interorbital line were parallel to the horizontal line and the angles were measured relative to these lines. Shewinvanakitkul et al. [27] used the mandibular plane as reference and reported its excellent reliability. Most relevant previous studies did not classify patients based on their sagittal skeletal pattern [23, 26, 34]. However, this was done in our study using the ANB angle and the Wits appraisal. The results showed a significant difference in inclination of right and left first and second molars between different sagittal skeletal patterns, such that the mean of these variables in class I and class III patients was significantly higher than that in class II patients. This finding may be due to the fact that in class II sagittal skeletal pattern, a wider part of the maxilla is positioned against a smaller part of the mandible. Thus, in order to compensate for the shortage in occlusal surface of the mandible, maxillary molars have palatal inclination (compared with class I and class III) and mandibular molars have buccal inclination. Thus, the inclination of molars of the maxilla and mandible is such that it compensates the occlusal surface shortage of the molars of the opposing jaw. On the other hand, the inclination of the right and left first and second molars of the mandible was significantly different among different sagittal skeletal patterns such that the mean of this variable in class II patients was significantly higher than that in class I and III patients. Thus, it may be stated that in case of reduction in occlusal surface area in the maxilla or mandible following an increase in inclination of molar teeth in a specific skeletal class, the opposing jaw would compensate the occlusal surface shortage by proper inclination of teeth. McNamaraa [35] stated that the position of mandibular teeth mainly depends on the shape of the opposing teeth in the maxillary arch rather than the shape of other mandibular teeth. Moreover, he added that the inclination of teeth may change in order to achieve an efficient position to maintain the integrity of dental arch.

With regard to the maxillary and mandibular right and left canine teeth, our results revealed no significant difference in inclination of teeth among different sagittal patterns. Shewinvanakitkul et al. [27] measured the buccolingual inclination of mandibular first molars in Thai patients with a mean age of 13.2 years using CBCT and reported that the inclination of first molars in class II patients was significantly lower than that in class I molar relationship. Their results were different from our findings, which may be due to differences in race and age of patients and the methodology of studies.

A previous study demonstrated that the buccolingual inclination of mandibular molars decreased from the anterior towards the posterior teeth. In other words, the lingual inclination of second molar was higher than that of first molar, which was in line with our findings [27]. In the present study, the buccolingual inclination of maxillary molars increased from the anterior towards the posterior region. In other words, the second molars had higher buccal inclination than the first molars.

Opinions of the experts are widely variable regarding the occlusal curve and rotation of molar teeth. Andrews [36] suggested the theory of the six keys, stating that the crown of each tooth should have an appropriate inclination in order for its occlusal surface to have an efficient function with the help of the occlusal surface of the opposing teeth. McNamaraa [37] suggested that flattening of the occlusal surface and the curve of Wilson should be included as a treatment goal in orthodontic treatment planning. In contrast, Dawson believed that when the curve of Wilson is excessively flattened, the masticatory function is impaired [38]. ABO suggests that the maxillary buccal cusps or the mandibular lingual cusps should not have more than 1 mm deviation from the vertical axis [15]. In general, it is suggested that in orthodontic treatment, the curve of Wilson should be maintained to the level that it does not impair the function of mastication [13]. According to Dawson [38], the curve of Wilson aims to achieve two goals: the first goal is to create an efficient position for maximum resistance against masticatory forces. In order to achieve this goal, the buccolingual inclination of posterior teeth should be parallel to the direction of applied load and orientation of internal pterygoid muscle. The second goal is that occlusal inclination enhances the access to food during the process of mastication. Okeson [10] explained that the curve of Wilson aims to create maximum intercuspation. Nanda [39] stated that the presence of the curve of Wilson between the buccal surfaces results in more effective occlusal function. Our results regarding the curve of Wilson showed that the mean of this variable in second molars of class II patients was significantly higher than that in class I patients and the mean of this curve in first molars was not significantly different among different sagittal skeletal patterns. In order to measure the curve of Wilson in this study, the mesiobuccal and mesiolingual cusp tips of the maxillary first and second molars along the buccal groove were connected to form an angle and this angle was measured. Thus, the smaller the difference in height of the buccal and lingual cusps of a tooth, and the higher the palatal inclination of that tooth, the larger the angle formed between the two opposing teeth and the flatter the curve would be (the smaller the curve of Wilson would be). Thus, the angle formed between the two opposing teeth had an inverse correlation with the curve of Wilson. In class II patients, considering the greater palatal inclination of molars compared with class I and class III, the angle between the maxillary second molars would be significantly higher and the curve of Wilson would be smaller. It is important to determine the ideal amount of this curve and buccolingual inclination of teeth for efficient function in different classes of occlusion. Such assessments can play a fundamental role in achieving the orthodontic treatment goals. Future studies are required to assess the correlation of curve of Spee and WALA-FA distance with the curve of Wilson in different sagittal skeletal patterns. Also, the inclination of molar and canine teeth should be investigated with regard to the inclination of their surrounding bone. Controlled studies are also recommended to assess the effect of age on tooth inclination and the curve of Wilson.

## 5. Conclusion

According to the results, maxillary molars have lower inclination and mandibular molars have higher inclination in class II sagittal pattern while no significant difference was noted in inclination of maxillary and mandibular canines in different sagittal skeletal patterns. The curve of Wilson in second molars of class II patients was significantly higher than that in class I. In different sagittal skeletal patterns, a compensatory relationship exists between the opposing teeth, which along with the standards of crowns, can be used to determine the appropriate position of teeth in dental arch.

## Figures and Tables

**Table 1:** Gender distribution based on sagittal skeletal pattern.

Cl 1		Cl 2		Cl 3	
Gender	Count	Column *N*%	Count	Column *N*%	Count	Column *N*%
Female	12	54.5	11	50.0	11	50.0
Male	10	45.5	11	50.0	11	50.0
Total	22	100.0	22	100.0	22	100.0

**Table 2:** Comparison of the inclination of teeth among the three sagittal skeletal patterns.

	Cl 1		Cl 2		Cl 3		*P* value
	Mean	SD	Mean	SD	Mean	SD	
Inclination of maxillary second molar. Right	104.77^b^	3.45	100.20^a^	5.21	103.53^b^	4.05	0.002^‡^
Inclination of maxillary first molar. Right	97.42^b^	3.63	92.27^a^	5.36	98.32^b^	1.89	<0.001^†^
Inclination of maxillary canine. Right	99.38^a^	3.40	91.80^a^	18.61	98.62^a^	3.95	0.053^‡^
Inclination of maxillary canine. Left	98.92^a^	3.74	96.76^a^	4.33	98.30^a^	2.95	0.149^‡^
Inclination of maxillary first molar. Left	97.97^b^	2.80	91.35^a^	6.08	97.84^b^	2.42	<0.001†
Inclination of maxillary second molar. Left	105.63^b^	3.40	101.77^a^	6.90	102.42^ab^	3.81	0.008†
Inclination of mandibular second molar. Left	70.43^a^	4.65	77.07^b^	4.74	72.87^a^	4.23	<0.001^‡^
Inclination of mandibular first molar. Left	81.64^a^	2.99	84.65^b^	3.05	82.57^a^	1.92	0.002^‡^
Inclination of mandibular canine. Left	95.98^a^	3.62	96.31^a^	4.73	95.61^a^	4.19	0.858^‡^
Inclination of mandibular canine. Right	97.19^a^	3.30	97.04^a^	5.56	96.10^a^	3.66	0.658^‡^
Inclination of mandibular first molar. Right	81.56^a^	2.96	84.83^b^	2.90	82.23^a^	1.78	<0.001^‡^
Inclination of mandibular second molar. Right	69.97^a^	4.81	77.44c	4.37	73.57^b^	4.10	<0.001^‡^

^‡^One-way ANOVA followed by Tukey's test. ^†^Welch one-way ANOVA test followed by Tukey's test. Means with the same superscripted letters are not significantly different (*P* > 0.05).

**Table 3:** Comparison of the curve of Wilson among the three sagittal skeletal patterns.

	Cl 1		Cl 2		Cl 3		*P* value^‡^
	Mean	SD	Mean	SD	Mean	SD	
Wilson curve of maxillary second molars	144.68^a^	6.75	151.09^b^	9.08	148.17^ab^	8.34	0.038
Wilson curve of maxillary first molars	167.99^a^	5.40	165.81^a^	6.42	165.08^a^	6.08	0.253

^‡^One-way ANOVA followed by Tukey's test. Means with the same superscripted letters are not significantly different (*P* > 0.05).

**Table 4:** Mean inclination of teeth in the three sagittal skeletal patterns.

					Mean	Standard deviation	Minimum	Maximum
Jaw	Maxilla	Sagittal skeletal pattern	Cl 1	inc7	105.20	3.41	97.63	113.19
				inc6	97.70	3.21	90.21	104.88
				inc3	99.15	3.54	90.67	106.49
			Cl 2	inc7	100.98	6.09	82.46	110.28
				inc6	91.81	5.69	79.48	99.64
				inc3	94.28	13.59	10.53	106.00
			Cl 3	inc7	102.97	3.93	97.93	112.42
				inc6	98.08	2.16	92.12	102.15
				inc3	98.46	3.45	89.84	106.82
			Total	inc7	103.05	4.91	82.46	113.19
				inc6	95.86	4.88	79.48	104.88
				inc3	97.30	8.56	10.53	106.82
	Mandible	Sagittal skeletal pattern	Cl 1	inc7	70.20	4.68	60.42	78.37
				inc6	81.60	2.94	75.11	86.41
				inc3	96.59	3.48	90.74	103.59
			Cl 2	inc7	77.25	4.51	69.30	87.94
				inc6	84.74	2.94	75.89	89.81
				inc3	96.68	5.12	86.82	106.18
			Cl 3	inc7	73.22	4.13	62.93	82.85
				inc6	82.40	1.84	78.58	87.73
				inc3	95.85	3.89	85.30	102.70
			Total	inc7	73.56	5.28	60.42	87.94
				inc6	82.91	2.93	75.11	89.81
				inc3	96.37	4.21	85.30	106.18

**Table 5:** Mean inclination of teeth based on demographic variables of patients.

		inc7		inc6		inc3	
		Standard deviation	Mean	Standard deviation	Mean	Standard deviation	Mean
*Age*	<30	87.71	14.62	89.16	7.56	97.57	4.46
	≥30	88.90	16.61	89.61	7.72	96.10	8.39

*Gender*	Female	87.37	14.92	88.85	7.49	97.01	3.96
	Male	89.30	16.34	89.96	7.77	96.65	8.81

*Sagittal skeletal pattern*	Cl 1	87.70	18.06	89.65	8.66	97.87	3.72
	Cl 2	89.12	13.07	88.28	5.74	95.48	10.28
	Cl 3	88.10	15.49	90.24	8.13	97.16	3.89

## Data Availability

The data are available, but we cannot share them due to patient privacy.
